# Mapping the Research Landscape of *Poria cocos* as a Medicinal and Edible Fungus: A Bibliometric Analysis from January 1990 to April 2026

**DOI:** 10.3390/ph19091421

**Published:** 2026-09-08

**Authors:** Yaqin Yang, Shiling Zhang, Wanwei Shi, Xuefang Li, Yaji Wang, Mingtong Cui, Xingxin Yang, Jie Yu

**Affiliations:** Yunnan Key Laboratory of Southern Medicine Utilization, College of Pharmaceutical Science, Yunnan University of Chinese Medicine, Kunming 650500, China; rc.yaqin0902@zcmu.edu.cn (Y.Y.); zhangshiling0306@163.com (S.Z.); vonvi7@163.com (W.S.); lixuef100@126.com (X.L.); 1256544060@qq.com (Y.W.); 1009503438@qq.com (M.C.)

**Keywords:** *Poria cocos*, medicinal and edible fungus, bibliometrics, network analysis, visualization

## Abstract

**Background:** *Poria cocos* is a medicinal and edible fungus widely used in traditional Chinese medicine and functional food development. However, the global research structure and thematic evolution of this field remain insufficiently characterized. This study aimed to map publication trends, collaboration networks, knowledge bases, and emerging research themes in *Poria cocos* research. **Methods:** Publications related to *Poria cocos* were retrieved from the Web of Science Core Collection from 1 January 1990 to 1 April 2026. Articles and reviews were included. Bibliometric and visualization analyses were performed using Microsoft Excel and an open-source Python 3.11.8 workflow (NetworkX 3.6.1 and Matplotlib 3.10.8). Co-occurrence analysis was used to count the frequency of co-occurrence of certain elements (e.g., countries, regions, institutions, etc.); cluster analysis was used to classify keywords; and burst analysis was used to identify research trends and hotspots. **Results:** A total of 1404 records were included. Publication output increased markedly after 2014 and peaked in 2025, whereas 2026 data were incomplete. China contributed the largest share of publications and occupied the central position in international collaboration networks. The *Journal of Ethnopharmacology* and *International Journal of Biological Macromolecules* were major publication venues. Keyword and co-citation analyses indicated a thematic shift from phytochemical characterization and resource studies toward polysaccharides, pharmacological mechanisms, network pharmacology, and gut microbiota. **Conclusions:** This bibliometric analysis provides an overview of the evolving research landscape of *Poria cocos*. Future studies should strengthen data-driven mechanistic validation, quality standardization, translational pharmacology, and clinical evidence generation.

## 1. Introduction

*Poria cocos* (Schw.) Wolf (PC) is a member of the Polyporaceae family, and its dried sclerotium has a long history of application as a traditional Chinese medicine (TCM) [1]. It was documented in the *Shennong Bencao Jing* for its therapeutic effects in excreting dampness, promoting diuresis, strengthening the spleen, and tranquilizing the heart [2]. Modern pharmacological studies have demonstrated that PC contains diverse bioactive constituents, including polysaccharides, triterpenoids, proteins, and organic acids [3,4,5,6]. These compounds exhibit multiple biological activities, such as antioxidant and immunomodulatory properties [7,8,9], which are beneficial for maintaining human intestinal ecology [10,11,12,13], protecting against alcoholic liver injury [14,15,16,17], and improving sleep quality [18,19], indicating broad prospects for the development of functional foods and therapeutic agents. In recent years, driven by increasing public awareness of health and growing market demand, research focusing on the pharmacological effects and large-scale cultivation techniques of PC has expanded substantially [20,21,22,23]. Consequently, a systematic review of this field is necessary to elucidate the current research status, identify core hotspots and evolutionary trajectories, and project future directions. Bibliometric analysis serves as an effective methodological approach to achieve these objectives.

Bibliometrics is an interdisciplinary field that applies mathematical and statistical methods to quantitatively analyze knowledge carriers [24]. By evaluating quantitative indicators such as publication output, authorship distributions, and keyword frequencies, it effectively delineates the current research landscape and core themes within specific domains [25]. Bibliometric mapping is commonly performed with dedicated tools such as CiteSpace [26] and VOSviewer [27,28]. To ensure full reproducibility on the cleaned dataset, the co-occurrence and co-citation networks, density maps, clustering, and burst analyses in the present study were constructed and visualized using an open-source Python workflow based on the NetworkX 3.6.1 and Matplotlib 3.10.8 libraries.

Given the extensive historical application of PC as an edible and medicinal fungus, alongside its contemporary economic value spanning from large-scale cultivation to health product development, a quantitative assessment of this field is warranted. This study retrieved the literature from the Web of Science Core Collection (WOSCC) database spanning approximately three decades (1990 to April 2026) using keywords such as “*Poria cocos*”, “Wolfiporia cocos”, and “Fuling”. Using a reproducible Python-based bibliometric workflow, the analysis was conducted, encompassing publication trends, contributing countries/regions, institutions, authors, keywords, and citation metrics. The systematic characterization of these data aims to clarify the research status, emerging hotspots, and future trajectories of PC, thereby providing an objective reference for subsequent scientific research and product development.

Although several narrative reviews have summarized the phytochemistry, pharmacology, and functional food potential of *Poria cocos*, a quantitative overview of its global knowledge structure, collaboration patterns, and thematic evolution remains limited. A bibliometric approach may complement traditional reviews by identifying influential contributors, knowledge bases, and emerging topics at a macro level. Therefore, this study aimed to characterize the publication trends, collaboration networks, core knowledge base, and thematic evolution of *Poria cocos* research using bibliometric and visualization methods.

## 2. Results

### 2.1. Analysis of the Number of Annual Publications

**Figure 1:**
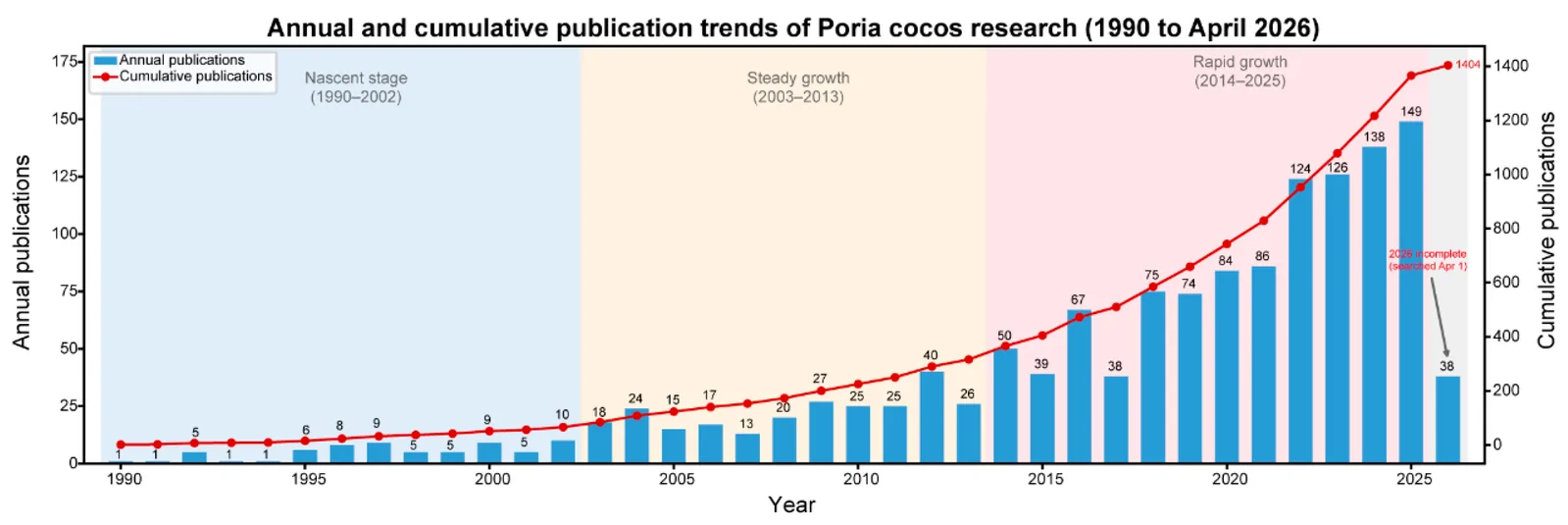
Analysis of the number of annual publications on PC from 1990 to April 2026.

A total of 1404 PC-related documents from the WOSCC database were analyzed (Figure 1). The specific publication years of these documents are presented in Table 1. The earliest literature on PC in this database dates back to 1990. The search was conducted on 1 April 2026; therefore, publication records for 2026 were incomplete and were not interpreted as a full-year trend. The overall trend of the number of publications is upward, but there are some local fluctuations. In the WOSCC database, the top three years for the number of publications were 2025 (149), 2024 (138), and 2023 (126). From 1990 to 1 April 2026, the annual publication output showed a sustained upward trend, particularly after 2014, while the apparent decrease in 2026 should be attributed to the incomplete coverage of that year. In summary, the sustained upward trend after 2014 suggests increasing academic interest in PC research.

### 2.2. Analysis of Subject Category Annual Publication

**Figure 2:**
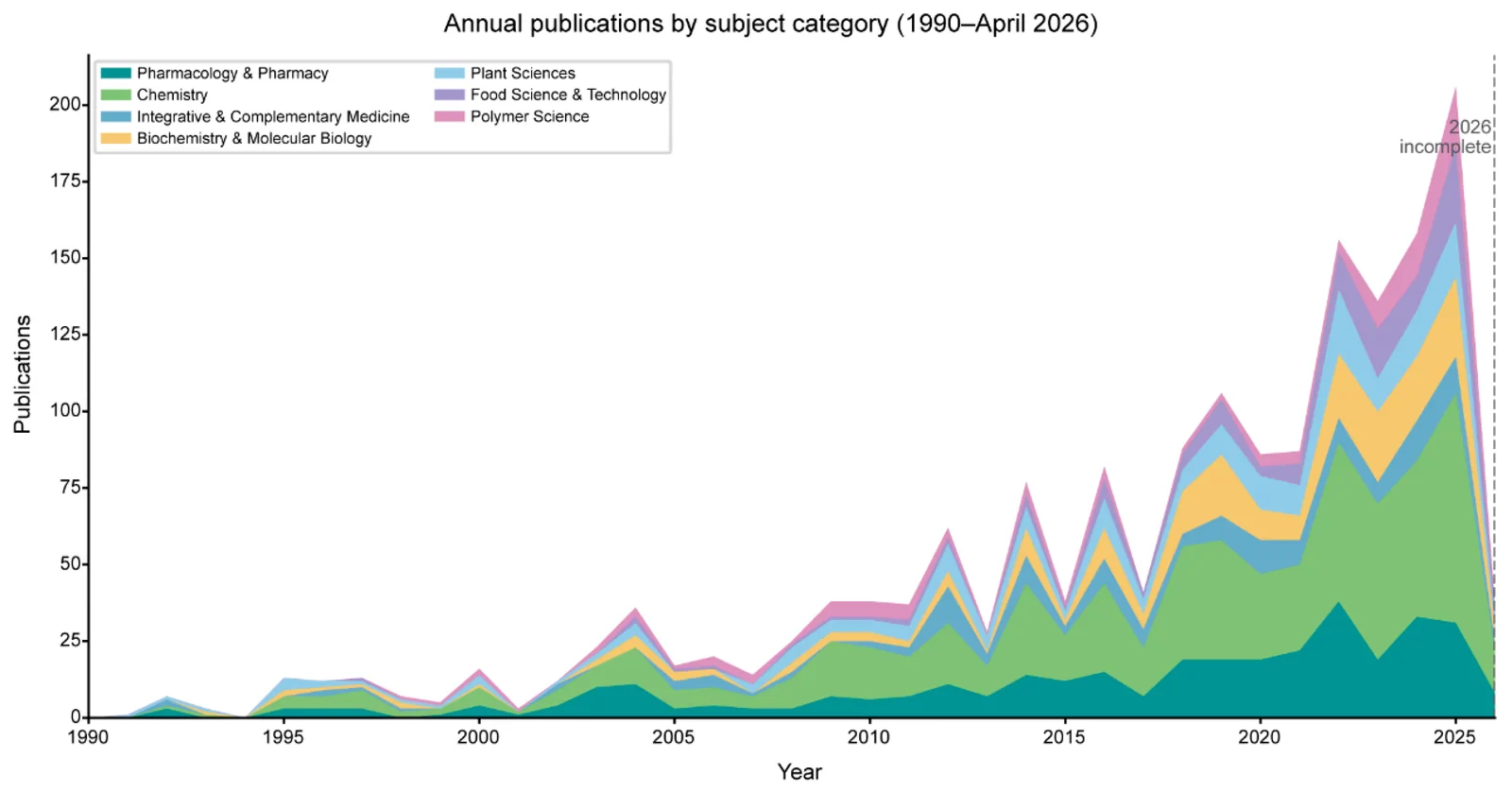
Annual publication trends across subject categories. Legend and axis labels were enlarged for readability.

Temporally, the stacked area chart maps the disciplinary expansion and structural shifts in PC research. Drawing on annual data from 1990 to April 2026 (Figure 2), the top seven subject categories form the core disciplinary landscape of this field, displaying heterogeneous growth dynamics across different periods.

It is noteworthy that the publication volumes across all subject categories in 2026 show a significant decline compared to 2025 (e.g., Pharmacology & Pharmacy from 37 to 9; Chemistry from 31 to 6; Integrative & Complementary Medicine to 6). This apparent decline is more likely attributable to database indexing delays and the incomplete nature of the current year’s data, rather than a genuine attenuation of research enthusiasm. Therefore, the 2026 data should be interpreted as a provisional observation. Ultimately, the subject category stack chart illustrates an evolutionary paradigm wherein PC research originated with pharmacy as the vanguard, followed by the continuous strengthening of chemistry and molecular biology, and culminating in the recent accelerated development of food science and engineering-related directions.

### 2.3. Analysis of Distributions of Countries/Regions

**Figure 3:**
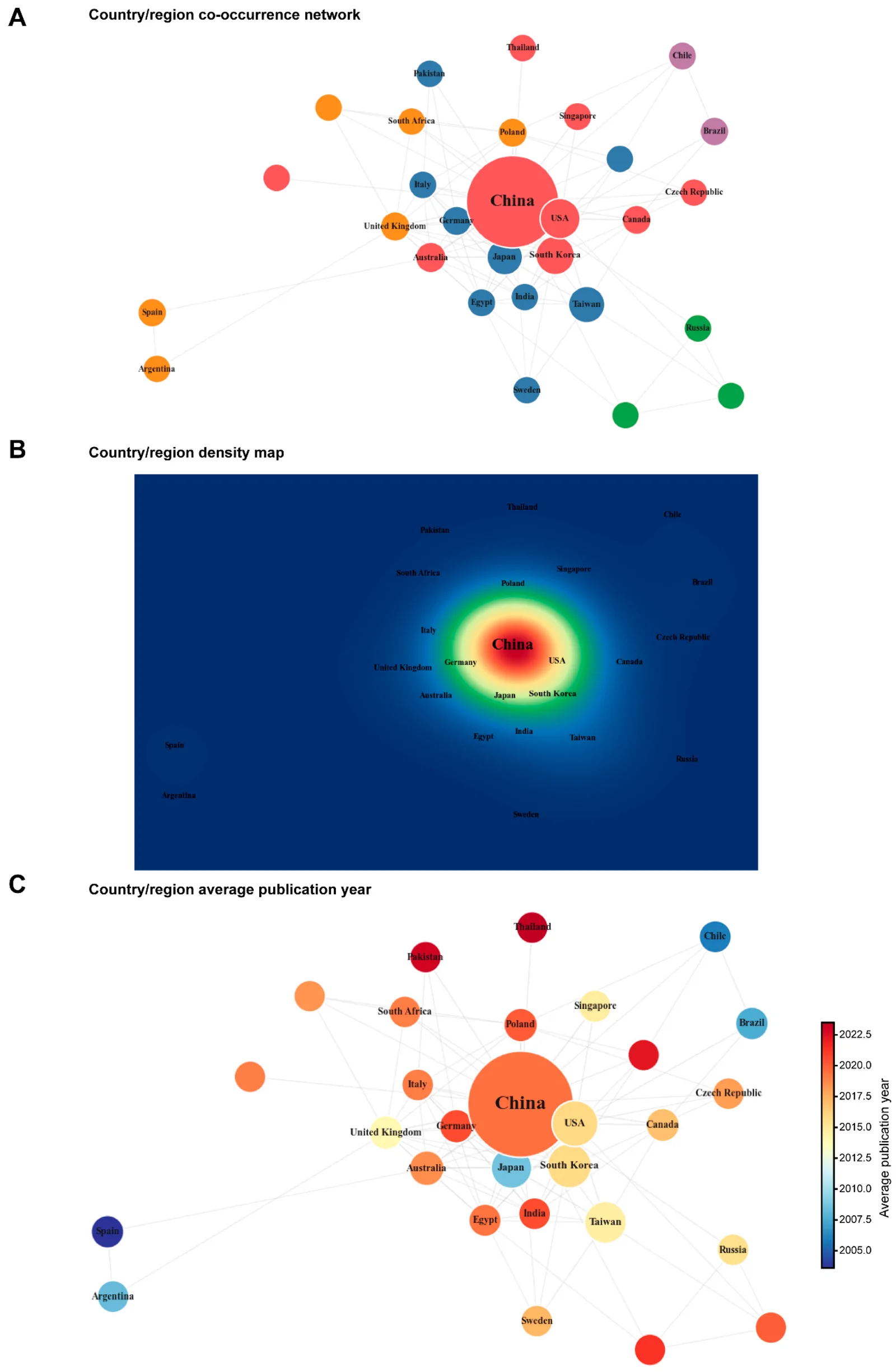
Country/region co-occurrence analysis: (**A**) network (Q = 0.1477), (**B**) density map, and (**C**) average publication year. In panel (**A**), nodes represent countries/regions, node size is proportional to publication count, node color indicates the Louvain community, and edges indicate co-occurrence links. In panel (**B**), the blue-to-red scale denotes relative local density from low to high. In panel (**C**), node color denotes average publication year. Major nodes are selectively labeled in panel (**A**) for readability.

The country/region co-occurrence network (Figure 3A) comprised 29 nodes and 99 links at the prespecified threshold, with modularity Q = 0.1477. China occupied the largest node and had links with several major collaborating countries, including the United States, Japan, Australia, South Korea, the United Kingdom, and Germany. These observations describe collaboration within the WOSCC-indexed dataset and should not be interpreted as a complete measure of global PC research activity.

The density distribution map (Figure 3B) further reinforces this feature. The region corresponding to China forms the most significant high-density core, with the US as a secondary hotspot. The areas surrounding Japan, South Korea, the UK, Australia, and Germany exhibit continuous but relatively weaker hotspots, demonstrating that international collaboration in this field is not completely discrete; rather, it presents a hierarchical clustering pattern driven by a few core countries.

The timeline overlay map (Figure 3C) reveals clearer phased evolutionary characteristics: the countries that participated in this field earlier mainly include Spain, Chile, Brazil, Argentina, and Japan, whose nodes predominantly appear blue or bluish-purple on the map. In contrast, countries that have been more active in recent years primarily include China, Germany, India, Poland, Saudi Arabia, Pakistan, Romania, Thailand, and Indonesia, with their node colors being noticeably warmer. Thus, it can be seen that the international collaboration landscape of PC research has experienced a transition from early scattered participation by a few countries to a multi-regional synergistic expansion centered on China. The collaboration vitality in recent years is mostly observed in Asia and certain newly participating countries. However, from the perspective of the overall network morphology, international collaboration still exhibits the structural characteristics of “high agglomeration among core countries, broad distribution of peripheral countries, and a gradient disparity in collaboration intensity.”

### 2.4. Analysis of Research Institutions

**Figure 4:**
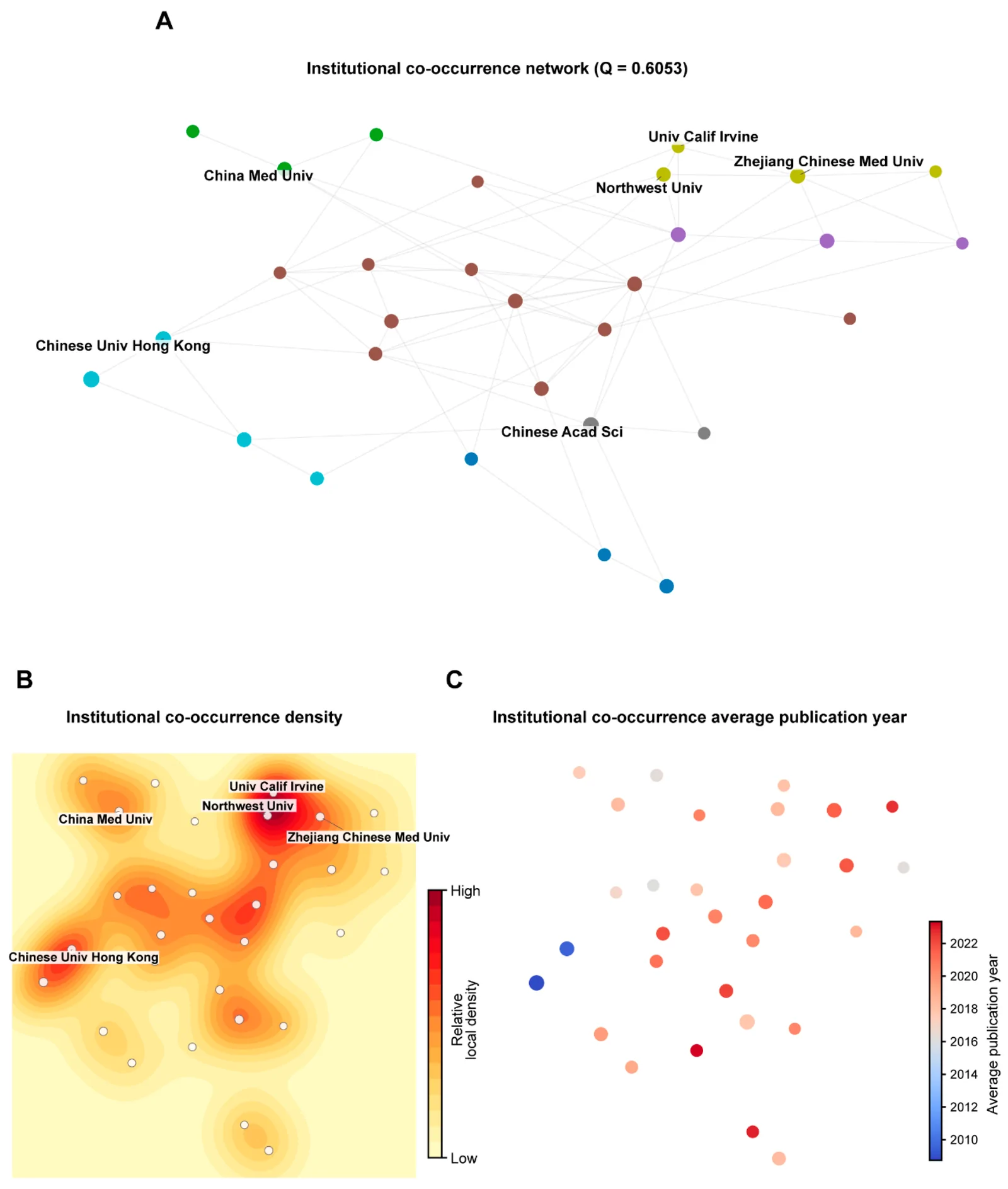
Institutional co-occurrence analysis: (**A**) network, (**B**) density, and (**C**) average publication year for the principal connected component (full network Q = 0.6053). In panel (**A**), nodes represent institutions, node size is proportional to publication count, node color indicates the Louvain community, and edges indicate co-occurrence links. In panel (**B**), yellow-to-dark-red indicates relative local density from low to high; five key institutions are labeled. In panel (**C**), node color denotes average publication year. Complete network tables are in Table S5.

The full institutional co-occurrence network comprised 37 nodes and 81 links, with modularity Q = 0.6053. Figure 4A displays its principal connected component, and Chinese Acad Sci, Wuhan Univ, Chinese Univ Hong Kong, Zhejiang Chinese Med Univ, and China Pharmaceut Univ were among the institutions with the largest publication counts. Major nodes are selectively labeled to preserve legibility; complete node and edge tables are supplied in Table S5.

The institutional density map (Figure 4B) visualizes areas of greater co-occurrence within the same principal connected component. The yellow-to-dark-red scale denotes relative local density from low to high, and five high-link-strength institutions are labeled as orientation points; complete node and edge tables are provided in Table S5.

The institutional timeline overlay (Figure 4C) displays the average publication year for the same principal connected component and is interpreted descriptively; it does not, by itself, establish temporal or causal mechanisms.

Table 2 details the data for the top 10 research institutions. In terms of publication volume, China boasts numerous major research institutions, with the top-ranked institutions occupying an overwhelming advantage in both publications and citations, highly consistent with the core nodes identified in the maps. Wuhan University (Wuhan Univ) ranks first with 47 publications, followed closely by the Chinese Academy of Sciences (46), which retains the highest total link strength and occupies the central position of the network. Synthesizing the map and table results, it is evident that the institutional collaboration network in this field not only sustains the foundational support of early high-impact institutions but also demonstrates a recent trend toward the integration of diverse institutional types and enhanced synergistic cooperation.

### 2.5. Analysis of Authors

**Figure 5:**
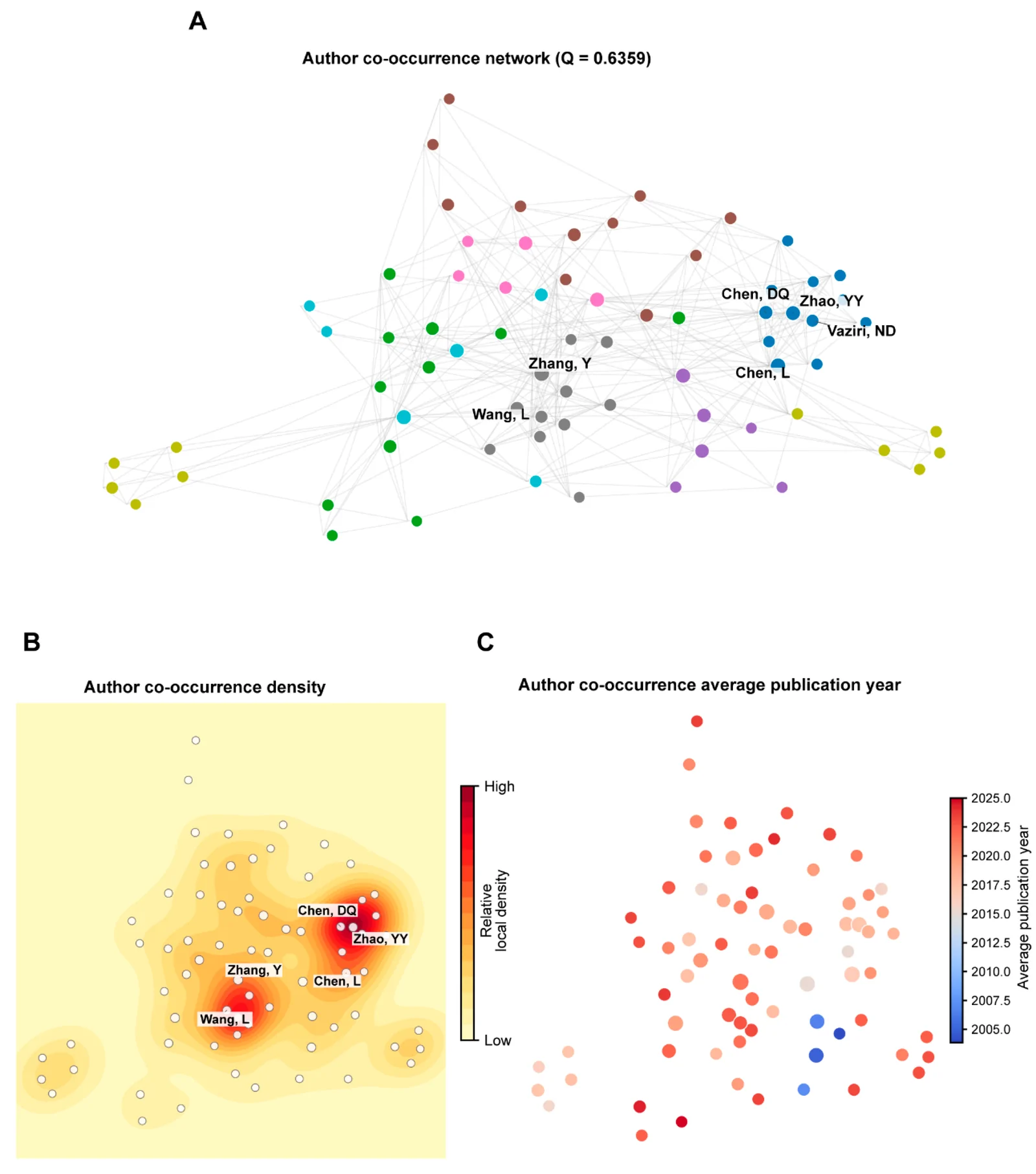
Author co-occurrence analysis: (**A**) network, (**B**) density, and (**C**) the average publication year for the principal connected component (full network Q = 0.6359). In panel (**A**), nodes represent authors, node size is proportional to publication count, node color indicates the Louvain community, and edges indicate co-authorship links. In panel (**B**), yellow-to-dark-red indicates relative local density from low to high; five key authors are labeled. In panel (**C**), node color denotes average publication year. Complete network tables are in Table S5.

The full author co-occurrence network comprised 179 nodes and 782 links, with modularity Q = 0.6359. Figure 5A displays its principal connected component, which contained multiple collaborative clusters and comparatively sparse links between some clusters. Only the most connected authors are labeled in the main panel to avoid text overlap; the complete author node and edge tables are supplied in Table S5.

The author density map (Figure 5B) visualizes areas of greater co-authorship co-occurrence within the same principal connected component. The yellow-to-dark-red scale denotes relative local density from low to high, and five high-link-strength authors are labeled as orientation points; complete node and edge tables are provided in Table S5.

The author timeline overlay (Figure 5C) displays the average publication year for the same principal connected component and is interpreted descriptively; it does not, by itself, establish temporal or causal mechanisms.

### 2.6. Analysis of Journals

**Figure 6:**
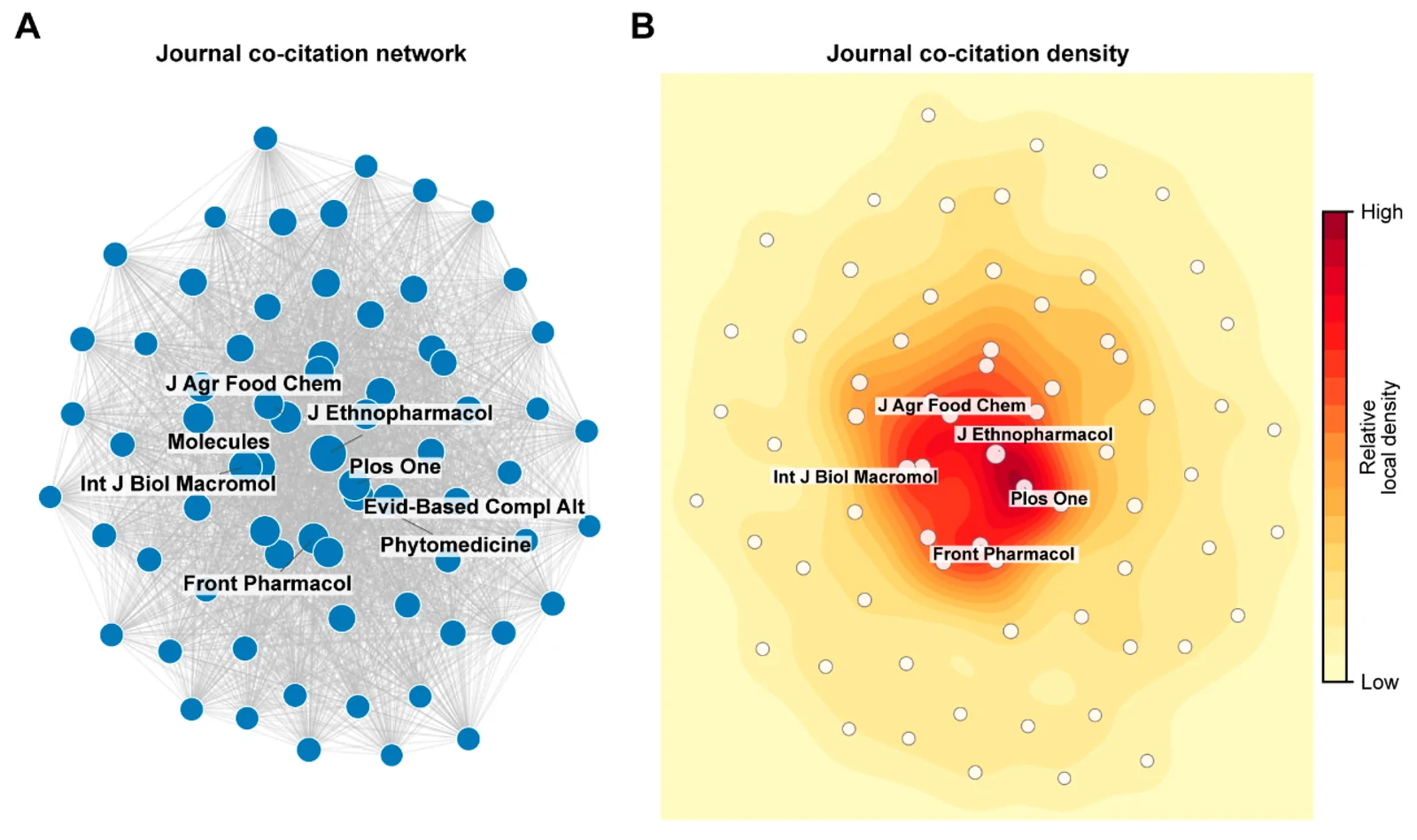
Journal co-citation analysis: (**A**) network and (**B**) density for the principal connected component. In panel (**A**), nodes represent journals and edges indicate co-citation links. In panel (**B**), the yellow-to-dark-red scale denotes relative local density from low to high; five key journals are labeled.

The journal co-citation network (Figure 6A) illustrates the knowledge-dissemination structure of the WOSCC-indexed dataset. To improve interpretability, the revised panel selectively labels major journals rather than displaying overlapping labels for every node. Publication-venue statistics are reported separately in Table 3.

The journal co-citation density map (Figure 6B) complements the selectively labeled principal connected component in Figure 6A. Its yellow-to-dark-red scale denotes relative local density from low to high, and five high-link-strength journals are labeled as orientation points; the network map and Ta-ble 3 provide the principal evidence for journal-level interpretation.

A further analysis combining publication and citation data reveals that different journals play differentiated roles in knowledge dissemination. On the one hand, certain journals with limited publication volumes exhibit exceptionally high average citation efficiencies. For instance, *Carbohydrate Polymers* (averaging 55.10 citations per paper) and *Phytomedicine* (averaging 33.47 citations per paper) demonstrate that seminal early-stage high-quality research was preferentially published in specialized core platforms dedicated to botany and polysaccharide analysis. On the other hand, journals such as the *Journal of Ethnopharmacology* and the *International Journal of Biological Macromolecules* have assumed the function of large-scale, conventional research output.

### 2.7. Analysis of References

**Figure 7:**
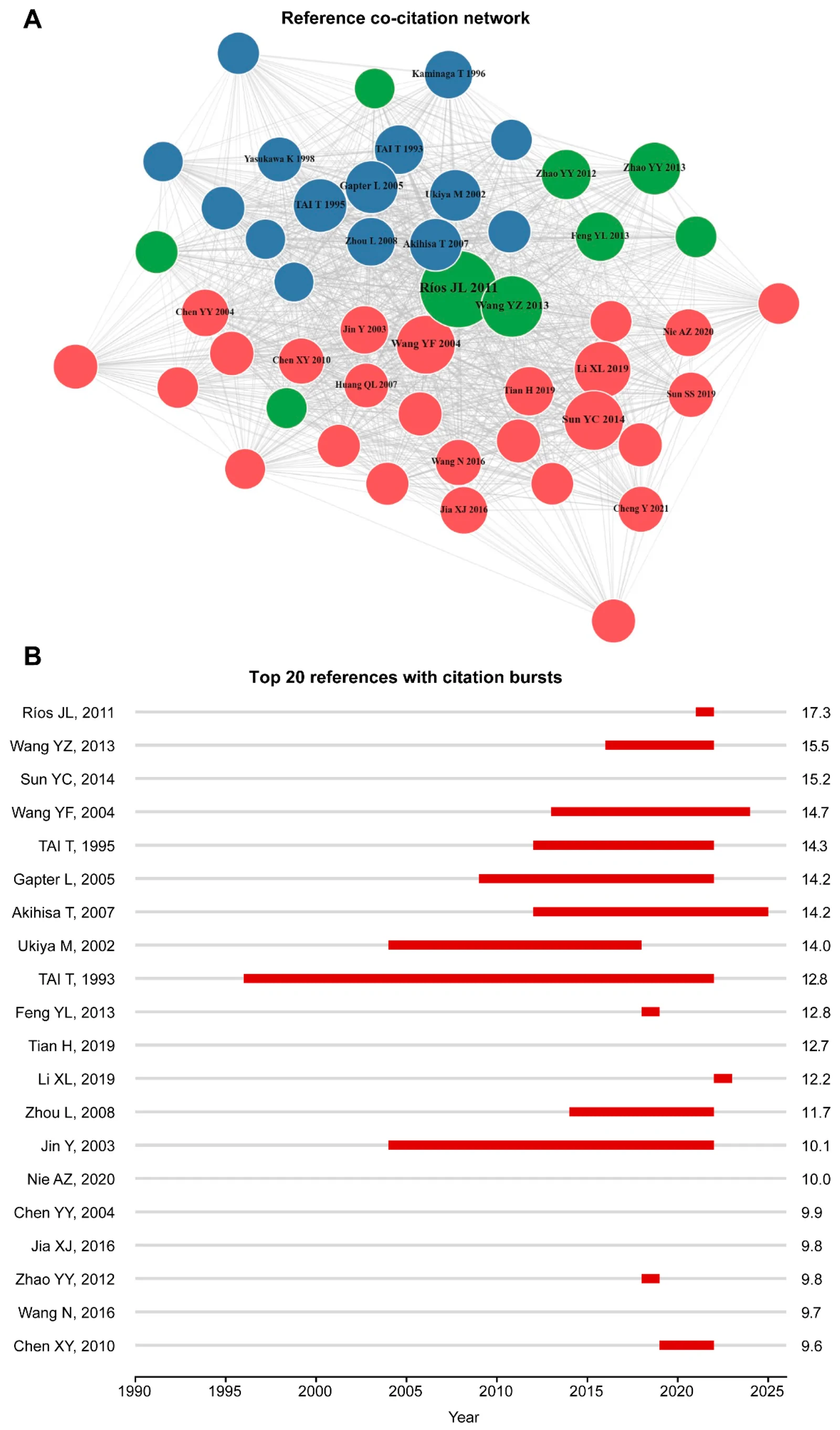
Analysis of references. (**A**) Reference co-citation network map, in which nodes represent references, node color indicates the co-citation cluster, and edges indicate co-citation links; (**B**) top 20 references with the strongest citation bursts (2000–2026). References in panel (**B**) are identified by first author and publication year, and red bars indicate the burst periods.

The document co-citation network (Figure 7A) reveals the classic origins and evolutionary trajectory of PC research from the perspective of the knowledge base. The network exhibits a prominent spatial layout characterized by a “left-right dual-plate” pattern: the right plate, dominated by blue-green nodes, aggregates early foundational literature that continues to be cited (e.g., Ríos JL, 2011 [29]); the left plate, dominated by orange-red nodes, is densely populated by the recently published literature with high co-citation frequencies. The presence of transitional connections between the two plates indicates that the current research frontier has expanded upon these early foundations. Regarding core node data, both the high-frequency co-cited and the high-degree literature show a significant shift toward the recently active zone on the left. Ríos JL (2011) [29] led with 176 co-citations, followed by Wang YZ (2013) [30] and Sun Y (2014) [21]; other frequently co-cited works included Akihisa T (2007) [31], Nie A (2020) [32], Li XL (2019) [33], and Tian H (2019) [34]. Furthermore, publications such as Lin TY (2020) [35] and Wu K (2019) [36] demonstrate outstanding network centrality, playing pivotal hub roles in bridging different research themes and integrating the recent knowledge network.

The reference burst map (Figure 7B) sensitively captures the phased evolutionary patterns of the knowledge base in PC research. Based on burst timing and intensity, this evolutionary trajectory can be delineated into three stages. The first stage (2007–2013) represents an early accumulation period, characterized by a scarcity of burst references (e.g., Akihisa T, 2008 [31]) primarily focused on basic compositional understanding. The second stage (2012–2020) is a rapid expansion period, featuring significantly increased and continuous burst bars, represented by Ríos JL (2011) [29] and Wang YZ (2013) [30], during which the research perspective deepened toward pharmacological effects and quality evaluation. The third stage (2020–present) marks a frontier agglomeration period, where the number of burst references further climbs with striking contemporaneity—among the top 20 high-burst references, several maintain their burst periods through to 2025.

An analysis of the publishing venues reveals that recently sustained burst references are highly concentrated in journals such as the *International Journal of Biological Macromolecules*, *Carbohydrate Polymers*, and *Frontiers in Pharmacology*. This spatiotemporal evolution clearly indicates that while early high-burst references primarily served to establish the foundational cognitive framework of the field, the current knowledge base has undergone a distinct thematic shift. The research frontier is now robustly converging on polysaccharides/macromolecular components, biological activity mechanisms, and the elucidation of pharmacological effects.

### 2.8. Analysis of Keywords

**Figure 8:**
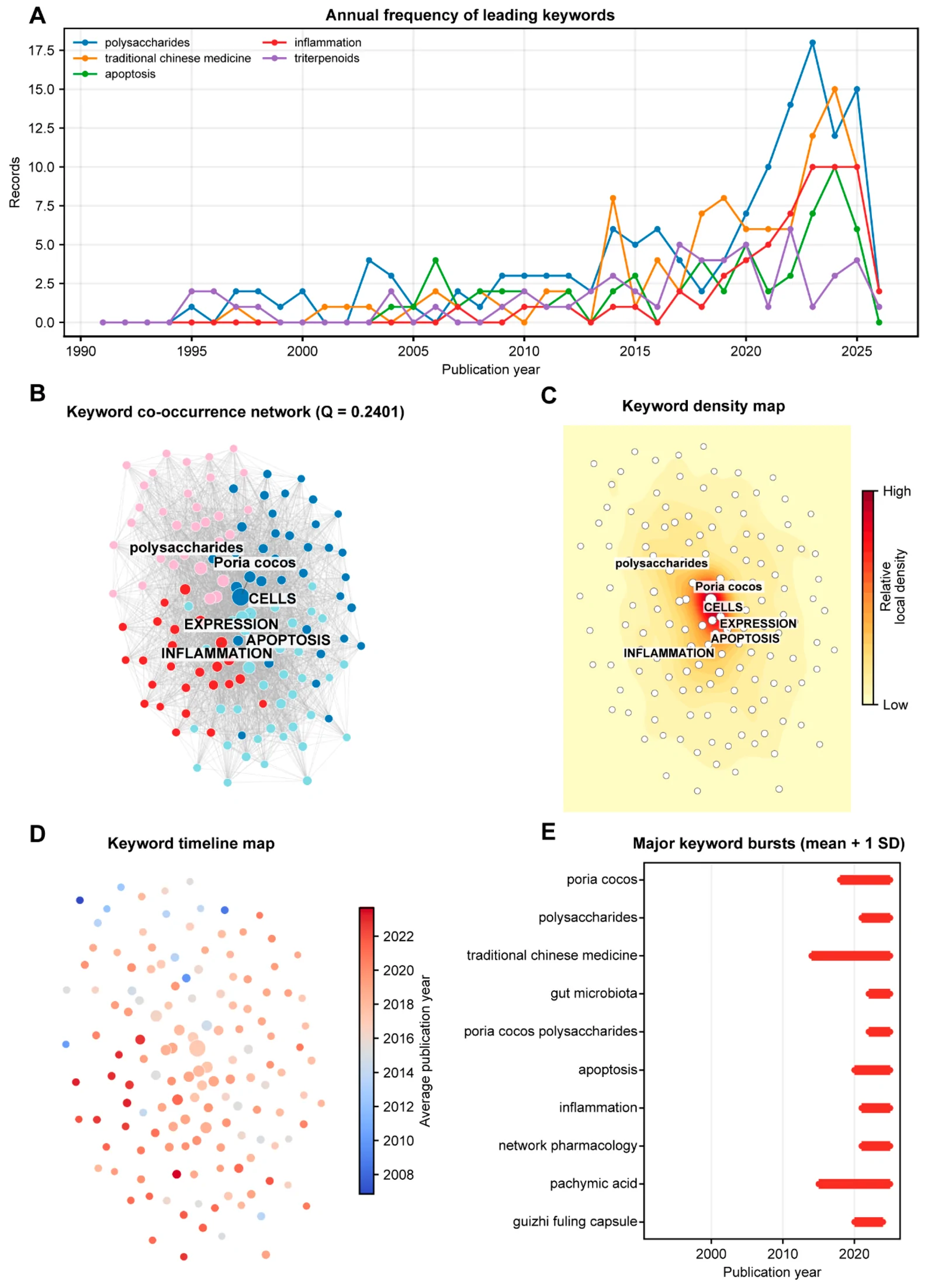
Keyword analysis: (**A**) annual publications by keyword; (**B**) co-occurrence network; (**C**) density map; (**D**) timeline map; and (**E**) major keyword bursts. Panels (**B**–**D**) show the high-link-strength principal connected component; complete network tables are in Table S5. In panel (**C**), yellow-to-dark-red indicates relative local density from low to high; six key terms are labeled.

The annual keyword distribution (Figure 8A), together with the co-occurrence, density, and timeline panels (Figure 8B–D), describes thematic structure and average temporal distribution in the WOSCC-indexed literature. In Figure 8C, yellow-to-dark-red represents relative local density from low to high, and six high-link-strength terms are retained as orientation labels. These results are descriptive and do not demonstrate that one topic caused the emergence of another.

A thematic-evolution (Sankey) analysis, available from the corresponding author on request, illustrates the continuity, differentiation, and recombination of research themes across time slices. The thematic-evolution network is anchored by PC, demonstrating a robust backbone continuity. As the network evolved, certain clinical, methodological, and component-related themes diverged, clearly mapping a phased trajectory that transitions from “basic compositional analysis” to “mechanism analysis, clinical evaluation, and interdisciplinary methodologies.”

The keyword-burst panel (Figure 8E) presents the descriptive mean-plus-one-standard-deviation heuristic specified in Section 2.7. It is interpreted as supporting trend information and not as a formal Kleinberg burst-detection result.

At the macroscopic clustering level, the keyword co-occurrence network contained 356 nodes and 8491 links at the prespecified threshold (modularity Q = 0.2401). The revised figure selectively labels highly connected terms to prevent overlap in the dense core. The clustering pattern is interpreted as a descriptive representation of thematic structure and evolution, rather than evidence of causal relationships among topics.

Finally, Figure 8B–D provides a readable overview of keyword co-occurrence, density, and average temporal distribution. For readability, these panels display the high-link-strength principal connected component; the complete 356-node network is documented in Table S5. The visualized core contains PC-related and peripheral thematic groups. These patterns are descriptive; they identify frequently connected research themes but do not establish that any theme drove the development of another.

## 3. Discussion

The increasing scholarly interest in PC is evidenced by a sustained expansion in its associated literature. By integrating bibliometric methods with network visualization techniques, this study provides a multidimensional quantitative analysis of the PC research landscape. Analyzing publication trends, disciplinary distribution, geographic and institutional contributions, core authors, active journals, references, and keywords, the research systematically maps the field’s evolutionary trajectory, active research themes, and potential future directions.

### 3.1. From Traditional Medicinal Use to Molecular Pharmacology

Based on the annual publication counts from 1990 to April 2026, the output showed a sustained upward trajectory with marked acceleration in the most recent period. Specifically, PC research remained in a nascent stage during the 1990s, transitioned into a phase of gradual growth from 2003 to 2013, and experienced a marked inflection point post-2014, with further escalation after 2022. These dynamics indicate that the field currently maintains a robust level of academic output and exhibits an evolutionary pattern of accelerated expansion following a prolonged period of knowledge accumulation.

The geographical and institutional analyses reveal a highly centralized, yet evolving, landscape within the WOSCC-indexed dataset. China occupied the hub position and had the highest total link strength. This pattern is consistent with substantial research activity surrounding PC in China; however, the bibliometric data do not establish historical use, biodiversity, or strategic investment as causal explanations. The apparent China-centered structure may also be affected by exclusive WOSCC coverage, which can underrepresent Chinese-language and locally indexed literature.

Institutionally, the shift from early comprehensive universities (like the Chinese Academy of Sciences and Wuhan University) to recently active universities of traditional Chinese medicine (such as Zhejiang Chinese Med Univ) reflects a maturation of the research paradigm. Early foundational efforts, primarily driven by comprehensive institutions, likely focused on basic physicochemical characterization and broad biological screening. The recent influx of specialized TCM universities indicates a disciplinary pivot towards deeper translational research, seeking to validate traditional medicinal knowledge through modern pharmacological and clinical frameworks. Furthermore, the exceptional citation performance of institutions like Northwest University, despite a relatively lower publication volume, highlights a critical insight: sheer output size does not equate to academic influence. This suggests that targeted, high-quality, and innovative research can generate disproportionate impact, challenging the volume-driven evaluation metrics and underscoring the necessity for institutions to cultivate specialized niches rather than merely expanding output. Moving forward, bridging the gap between comprehensive research hubs and specialized TCM institutions, alongside fostering multi-polar international collaborations beyond the current core-periphery structure, will be vital for the sustainable and multidimensional advancement of PC research.

The author collaboration network reveals a “multi-core parallel” paradigm in PC research, characterized by robust intra-group connectivity but insufficient inter-group coupling. While highly cohesive teams ensure research depth within specific niches, the sparse cross-cluster connections indicate a prominent “silo effect” that may hinder the cross-pollination of ideas. Nevertheless, the recent outward radiation of new authors from early cores to emerging clusters signals a dynamic expansion. To accelerate breakthroughs, future efforts should prioritize fostering cross-team collaborations to dismantle academic islands and promote knowledge integration across the field.

The journal co-occurrence network highlights a differentiated knowledge dissemination system in PC research, with the *Journal of Ethnopharmacology* acting as its undisputed core. Functioning as the primary academic hub, it bridges the critical gap between traditional medicine and modern pharmacological validation. While the *Journal of Ethnopharmacology* and the *International Journal of Biological Macromolecules* dominate as high-volume platforms driving the field’s mainstream output, specialized journals like *Carbohydrate Polymers* and *Phytomedicine* exhibit exceptional per-paper citation efficiency, preserving the foundational knowledge of early polysaccharide chemistry. This dual structure indicates that the field’s evolution relies on both the massive, cross-disciplinary convergence anchored by ethnopharmacology and the deep, high-impact contributions from niche chemical platforms. Moving forward, enhancing knowledge flow between these high-volume ethnopharmacological hubs and specialized chemistry journals will be vital for translating basic structural insights into modern therapeutic applications.

### 3.2. Methodological Shift from Component Isolation to Systems Pharmacology

The knowledge base, comprising the highly cited literature within a specific domain, facilitates researchers’ comprehension of the foundational concepts guiding new scientific inquiries. The literature exhibiting high citation frequencies inherently possesses substantial academic value and disciplinary influence.

Reference burst analysis serves as a sensitive indicator for identifying knowledge bases that undergo rapid, intensive focus within defined periods, thereby elucidating phased hotspot transitions and frontier evolution. A review by Rios JL (2011), published in *Planta Medica* [29], exhibited the highest burst intensity (22.54) from 2012 to 2016, representing the most pronounced periodic knowledge expansion in the dataset. By systematically summarizing the chemical constituents and pharmacological properties of PC, this work directed subsequent research trajectories. Notably, 11 of the top 30 burst references maintain active burst periods extending to 2025, comprising over one-third of the analyzed documents. This temporal distribution indicates that the knowledge frontier of PC research remains highly dynamic.

Co-citation analysis further delineates the structural evolution of this knowledge base. Spatial mapping of the co-citation network reveals that the high-frequency co-cited literature is predominantly clustered within recently formed core nodes, including studies by Li XL (2019) [2], Cheng Y (2021) [37], Tian H (2019) [34], and Xu TR (2022) [38]. This clustering signifies a distinct shift in the knowledge base toward contemporary research findings. Among the top ten most cited papers, the antitumor, hepatoprotective, and immunomodulatory properties of PC have garnered significant attention. The sustained citation bursts of these recent publications extending to 2025 underscore the rapid turnover rate of the current knowledge base and the continuous integration of the cutting-edge literature into the core citation network.

### 3.3. Translational Gaps in PC Research

Keyword co-occurrence and clustering analyses identify pachymic acid, polysaccharides, network pharmacology, gut microbiota, apoptosis, and inflammation as prominent topics. Pachymic acid and triterpenoid themes mainly reflect constituent identification and preclinical mechanism-oriented research, whereas polysaccharide-related themes span structure–activity studies and preclinical pharmacology. Network pharmacology, molecular docking, and gut microbiota themes are hypothesis-generating or mechanistic directions, rather than clinical validation. This interpretation highlights the need for standardization, pharmacokinetic assessment, safety evaluation, and well-designed clinical studies.

It should be emphasized that network pharmacology, molecular docking, and related computational approaches are essentially hypothesis-generating; their predictions require rigorous experimental and clinical validation before mechanistic or therapeutic conclusions can be drawn. Several translational gaps also remain in PC research. First, the active constituents are not yet adequately standardized, as the structure, molecular weight, and bioactivity of PC polysaccharides and triterpenoids vary considerably with geographical origin, processing, and extraction methods. Second, evidence on pharmacokinetics and bioavailability—particularly for macromolecular polysaccharides and triterpenoids—remains limited. Third, high-quality clinical evidence is scarce, as most studies are still confined to in vitro systems, animal models, or computational prediction. Fourth, the development of PC-based functional foods requires systematic safety, quality-control, and dose–response data. Finally, international collaboration remains highly China-centered, which may constrain the cross-regional generalizability of the current findings.

Temporal sequence and burst analyses of keywords further illustrate that PC research has transitioned from an early knowledge structure primarily centered on taxonomic classification, component identification, and basic bioactivity into a multidimensional landscape where component research, mechanistic elucidation, TCM formulation applications, and data-driven methodologies advance concurrently. Specifically, the intersection of “network pharmacology, molecular docking, gut microbiota, and specific disease mechanisms” represents a highly active trajectory. The emergence of novel keywords post-2020 indicates that in-depth pharmacological mechanistic dissection and the application of comprehensive methodological frameworks will constitute the core frontiers of future research.

### 3.4. Future Research Agenda and Methodological Limitations

While this study provides a reproducible bibliometric characterization of WOSCC-indexed PC literature, several limitations should be acknowledged. First, WOSCC-only retrieval may underrepresent Chinese-language and locally indexed literature and may affect apparent geographic and collaboration structures. Second, the custom Python workflow was designed for transparent processing, but its clusters and burst outputs were not formally cross-validated against VOSviewer or CiteSpace. The burst heuristic is not equivalent to Kleinberg burst detection. Third, the threshold-sensitivity analysis showed that node counts, links, modularity, and connected components varied across adjacent thresholds (Supplementary Table S4), so the reported maps should be interpreted as threshold-dependent descriptive representations. Finally, the 2026 data represent an incomplete year and may be affected by indexing latency. Future work should use harmonized, deduplicated Scopus and PubMed/NCBI exports; PubChem may complement chemical interpretation but is not a bibliographic database.

## 4. Materials and Methods

### 4.1. Data Source

Literature data were retrieved exclusively from the Web of Science Core Collection (WOSCC) on 1 April 2026. The study was deliberately limited to WOSCC-indexed literature. After document-type and language screening, the supplied WOSCC export contained 1677 records. Title, abstract, and, where necessary, full-text screening removed 273 false-positive records, predominantly records in which “Fuling” referred to a geographic location or a shale-gas field, leaving 1404 records for analysis. WOSCC-only coverage may underrepresent the literature indexed elsewhere, including Chinese-language or locally indexed records; the results should therefore not be interpreted as a complete map of all global PC research. The complete list of included WOSCC records is provided in Table S6.

### 4.2. Search Strategy

The WOSCC Topic-field query was: TS = (“*Poria cocos*” OR Fuling OR “Fu Ling” OR Tuckahoe OR “Wolfiporia cocos” OR “Wolfiporia cocos (F. A. Wolf) Ryvarden & Gilb.” OR “Wolfiporia extensa” OR “Wolfiporia extensa (Peck) Ginns”). The Topic field covers title, abstract, author keywords, and Keywords Plus. The complete query and screening audit are provided in Supplementary Table S1.

### 4.3. Time Span

The search was performed on 1 April 2026 and covered records published from 1 January 1990 to 1 April 2026. Records indexed in 2026 were treated as incomplete and were not interpreted as a full-year trend.

### 4.4. Inclusion and Exclusion Criteria

**Figure 9:**
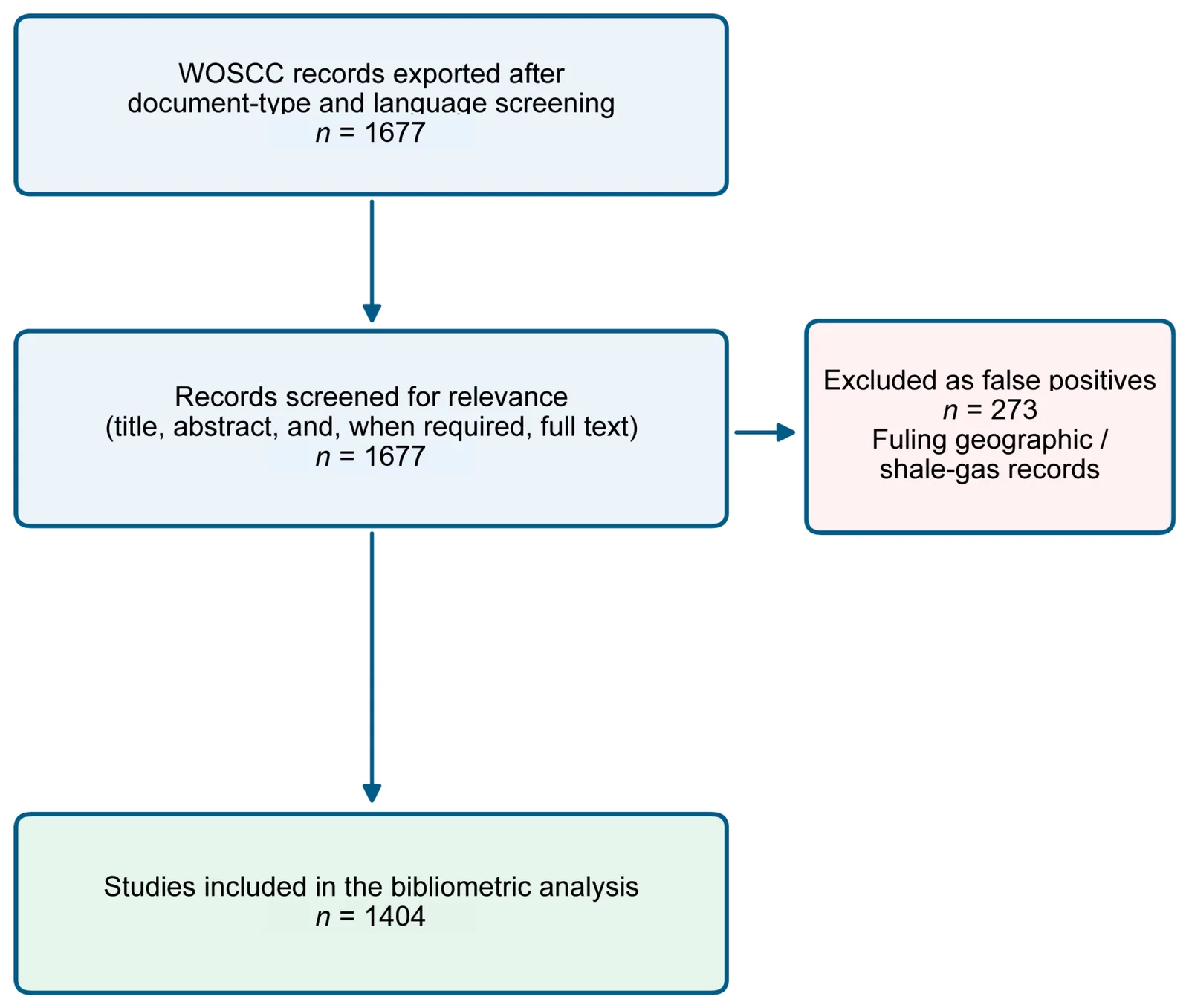
Study-selection flow based on the supplied WOSCC export: 1677 records after document-type and language screening, 273 documented false positives excluded, and 1404 records included.

Records with Article or Review as the primary document type and published in English or Chinese were eligible. Secondary Web of Science tags (e.g., Early Access, Proceedings Paper, Book Chapter, Data Paper, or Retracted Publication) were retained only when the primary type was Article or Review. The 1677 supplied records were screened for relevance. A documented list of 273 false positives, predominantly shale-gas and other geographic uses of “Fuling”, was excluded. The final WOSCC-indexed analysis set comprised 1404 records. Retraction status was not used as an additional exclusion criterion because bibliometric indicators were interpreted as descriptive rather than as evidence of research quality. The study-selection and screening workflow is shown in Figure 9.

### 4.5. Data Standardization

To ensure data consistency and yield more precise outcomes, synonym consolidation was performed prior to the analysis. This was achieved by applying a custom synonym thesaurus that merged variant spellings and abbreviations (e.g., *Poria cocos*/*Wolfiporia cocos*/PC) before network construction; the full thesaurus is provided in Supplementary Table S2.

### 4.6. Research Software

Bibliometric networks and visualizations were generated with an open-source Python 3.11.8 workflow. Microsoft Excel 2019 was used to organize the dataset and to plot annu-al-publication and subject-category trends. Co-occurrence networks (countries/regions, institutions, authors, and keywords) and co-citation networks (journals and references) were constructed using NetworkX 3.6.1; total link strength (TLS) was computed as the sum of a node’s co-occurrence/co-citation link weights, and node communities were detected by the Louvain community-detection implementation in NetworkX 3.6.1. Network maps, density maps, timeline overlays, and citation-burst charts were rendered with Matplotlib 3.10.8. Complete software and parameter settings are provided in Supplementary Table S3.

### 4.7. Statistical Analysis and Visualization

Network layouts were computed with a force-directed (Fruchterman–Reingold) algorithm. Node size was scaled to occurrence or citation frequency, edge width to link weight, and node color to the detected community or average publication year. The prespecified minimum-frequency thresholds were countries ≥ 3, institutions ≥ 12, authors ≥ 6, and keywords ≥ 6. Threshold sensitivity was assessed at adjacent thresholds (countries 2–4; institutions 10, 12, and 15; authors 5, 6, and 8; keywords 5, 6, and 8); the resulting node counts, links, modularity values, clusters, and connected components are reported in Supplementary Table S4. Citation and keyword bursts were identified using annual frequency exceeding the item-specific mean by more than one standard deviation. This transparent heuristic is descriptive and is not equivalent to the Kleinberg burst-detection algorithm used in CiteSpace.

## 5. Conclusions

This bibliometric analysis mapped the publication trends, collaboration networks, knowledge base, and thematic evolution of *Poria cocos* research from 1990 to April 2026. The field showed sustained growth, with China playing a central role in publication output and international collaboration. Research themes have gradually shifted from resource identification and phytochemical characterization toward polysaccharides, pharmacological mechanisms, network pharmacology, and gut microbiota. Future studies should emphasize standardized quality control, experimental validation of predicted mechanisms, translational pharmacology, and clinical evidence generation.

## Figures and Tables

**Table 1:** Annual publications and cumulative publications related to *Poria cocos* from 1990 to April 2026.

Year	Number of Publications	Cumulative Number of Publications
1990	1	1
1991	1	2
1992	5	7
1993	1	8
1994	1	9
1995	6	15
1996	8	23
1997	9	32
1998	5	37
1999	5	42
2000	9	51
2001	5	56
2002	10	66
2003	18	84
2004	24	108
2005	15	123
2006	17	140
2007	13	153
2008	20	173
2009	27	200
2010	25	225
2011	25	250
2012	40	290
2013	26	316
2014	50	366
2015	39	405
2016	67	472
2017	38	510
2018	75	585
2019	74	659
2020	84	743
2021	86	829
2022	124	953
2023	126	1079
2024	138	1217
2025	149	1366
2026	38	1404

**Table 2:** Top 10 institutions related to *Poria cocos*.

Rank	Institution	TLS	Publications	Total Citations	Avg. Article Citations
1	Wuhan Univ	57	47	1880	40.00
2	Chinese Acad Sci	91	46	1091	23.72
3	Chinese Univ Hong Kong	75	38	2223	58.50
4	Zhejiang Chinese Med Univ	66	38	1896	49.89
5	China Pharmaceut Univ	49	34	982	28.88
6	Guangzhou Univ Chinese Med	90	33	621	18.82
7	Nanjing Univ Chinese Med	54	32	623	19.47
8	Beijing Univ Chinese Med	70	31	612	19.74
9	Huazhong Agr Univ	60	30	522	17.40
10	Northwest Univ	59	29	2780	95.86

Note: Institution names are displayed in their standardized Web of Science short forms (e.g., Chinese Acad Sci, Chinese Academy of Sciences; China Pharmaceut Univ, China Pharmaceutical University; Zhejiang Chinese Med Univ, Zhejiang Chinese Medical University). TLS, total link strength.

**Table 3:** Top 10 journals publishing research on *Poria cocos*.

Rank	Journal	TLS	Publications	Total Citations	Avg. Citations
1	*Journal Of Ethnopharmacology*	613	94	3421	36.39
2	*International Journal Of Biological Macromolecules*	645	57	2014	35.33
3	*Evidence-Based Complementary And Alternative Medicine*	162	40	691	17.28
4	*Molecules*	378	33	612	18.55
5	*Frontiers In Pharmacology*	320	32	466	14.56
6	*Phytomedicine*	169	32	1071	33.47
7	*Carbohydrate Polymers*	335	30	1653	55.10
8	*American Journal Of Chinese Medicine*	135	21	378	18.00
9	*Journal Of Agricultural And Food Chemistry*	203	20	659	32.95
10	*Medicine*	37	18	96	5.33

## Data Availability

The processed data supporting the findings of this study are provided in the Supplementary Materials, including the search strategy, study-selection summary, included-record list, synonym thesaurus, and software/parameter settings. The raw Web of Science Core Collection export used in this study was obtained from Clarivate Analytics under licence and cannot be publicly redistributed by the authors. Access to the underlying database is available through the Web of Science Core Collection, subject to the applicable licence terms.

## Supplementary Materials

The following supporting information can be downloaded at: https://www.mdpi.com/article/10.3390/ph19091421/s1, Table S1. Search strategy and study selection. Table S2. Synonym thesaurus (terms merged before network construction). Table S3. Software and parameter settings. Table S4. Threshold-sensitivity analysis for country, institution, author, and keyword networks. Table S5. Threshold-sensitivity analysis and complete node and edge tables for country, institution, author, and keyword networks (provided as a separate Excel file). Table S6. Included WOSCC records (n = 1404; provided as a separate Excel file).

## Abbreviations

PC	*Poria cocos* (Schw.) Wolf
TCM	traditional Chinese medicine
WOSCC	Web of Science Core Collection
US	United States
UK	United Kingdom
TLS	Total Link Strength
