# Supplementary material for: Mapping the Research Landscape of Poria cocos as a Medicinal and Edible Fungus: A Bibliometric Analysis from January 1990 to April 2026
Source: Pharmaceuticals (Basel). 2026 Sep 8;19(9):1421. doi: 10.3390/ph19091421 (PMC13610884; doi:10.3390/ph19091421)
Supplement: Supplementary file 1 [file pharmaceuticals-19-01421-s001.zip › Supplementary_Materials_revised.pdf]

**Supplementary Materials**

Mapping the research landscape of Poria cocos as a medicinal and edible fungus: a bibliometric analysis from January 1990 to April 2026.

**Table S1. Search strategy and study selection**

| Database | Web of Science Core Collection (WOSCC) |
|---|---|
| Indexes | SCI-EXPANDED, SSCI, A&HCI, ESCI |
| Search field | Topic (TS): title, abstract, author keywords, and Keywords Plus |
| Search query | TS=("Poria cocos" OR Fuling OR "Fu Ling" OR Tuckahoe OR "Wolfiporia cocos" OR "Wolfiporia cocos (F. A. Wolf) Ryvarden & Gilb." OR "Wolfiporia extensa" OR "Wolfiporia extensa (Peck) Ginns") |
| Search date and timespan | Searched April 1, 2026; 1990-01-01 to 2026-04-01 |
| Languages | English and Chinese |
| Document types included | Article or Review as the primary WOS document type |
| Records after document-type and language screening | 1,677 |
| False positives removed after relevance screening | 273, predominantly Fuling geographic/shale-gas records |
| Records included in the bibliometric analysis | 1,404 |
| Records screened for relevance | 1,677 |
| False positives excluded after manual relevance screening | 273 |
| Records included in the bibliometric analysis | 1,404 |

Table S2. Synonym thesaurus (terms merged before network construction).

| Label | Replace by |
|---|---|
| poria-cocos | Poria cocos |
| poria cocos (schw.) wolf | Poria cocos |
| wolfiporia cocos | Poria cocos |
| wolfiporia extensa | Poria cocos |
| wolfiporia cocos (f.a. wolf) ryvarden & gilb | Poria cocos |
| wolfiporia cocos (f. a. wolf) ryvarden & gilb | Poria cocos |
| wolfiporia cocos (schwein.) ryvarden & gilb | Poria cocos |
| fu ling | Poria cocos |
| fu-ling | Poria cocos |
| hoelen | Poria cocos |
| tuckahoe | Poria cocos |
| poria cocos polysaccharide | Poria cocos polysaccharides |
| poria cocos polysaccharides | Poria cocos polysaccharides |
| pcp | Poria cocos polysaccharides |
| poria cocos polysaccharide (pcp) | Poria cocos polysaccharides |
| poria cocos polysaccharides (pcp) | Poria cocos polysaccharides |
| polysaccharide | polysaccharides |

| | |
|---|---|
| triterpene | triterpenoids |
| triterpenes | triterpenoids |
| triterpenoid | triterpenoids |
| anti-oxidant | antioxidant activity |
| antioxidant | antioxidant activity |
| chinese medicine | traditional Chinese medicine |
| chinese herbal medicine | traditional Chinese medicine |
| herbal medicine | traditional Chinese medicine |
| tcm | traditional Chinese medicine |
| traditional chinese medicine (tcm) | traditional Chinese medicine |
| intestinal microbiota | gut microbiota |
| gut microbiome | gut microbiota |
| intestinal flora | gut microbiota |
| gut microbiome | gut microbiota |
| intestinal microflora | gut microbiota |
| network pharmacology analysis | network pharmacology |
| net work pharmacology | network pharmacology |
| alzheimer disease | Alzheimer's disease |
| alzheimer's disease (ad) | Alzheimer's disease |
| ad | Alzheimer's disease |
| guizhi fuling wan | Guizhi Fuling capsule |
| guizhi-fuling capsule | Guizhi Fuling capsule |
| gui-zhi-fu-ling | Guizhi Fuling capsule |
| guizhi fuling formula | Guizhi Fuling capsule |
| poricoic acid a | poricoic acid A |
| in-vitro | in vitro |
| in-vivo | in vivo |

Table S3. Software and parameter settings.

| Python 3.11.8 | Overall bibliometric workflow. |
|---|---|
| Microsoft Excel 2019 | Dataset organization; annual-publication and subject-category trend charts. |
| NetworkX 3.6.1 | Construction of co-occurrence (countries/regions, institutions, authors, keywords) and co-citation (journals, references) networks; total link strength (TLS) = sum of a node link weights; force-directed (Fruchterman-Reingold) layout. |
| Matplotlib 3.10.8 | Rendering of network maps, density maps, timeline overlays, stacked-area charts, and citation-burst charts. Bursts: years exceeding an item mean annual frequency by >1 SD. |
| Threshold sensitivity analysis | Countries: 2–4; institutions: 10, 12, 15; authors: 5, 6, 8; keywords: 5, 6, 8. Node counts, links, modularity, clusters, and components are reported in Table S4. |

Table S4. Threshold-sensitivity analysis for network construction.

| Network | Minimum | Nodes | Links | Modularity | Louvain | Connected |
|---|---|---|---|---|---|---|

| | frequency threshold | | | Q | clusters | components |
|---|---|---|---|---|---|---|
| Country/region co-occurrence | 2 | 39 | 127 | 0.1879 | 6 | 1 |
| Country/region co-occurrence | 3 | 29 | 99 | 0.1477 | 5 | 1 |
| Country/region co-occurrence | 4 | 24 | 81 | 0.1338 | 4 | 1 |
| Institutional co-occurrence | 10 | 49 | 122 | 0.6215 | 9 | 3 |
| Institutional co-occurrence | 12 | 37 | 81 | 0.6053 | 8 | 2 |
| Institutional co-occurrence | 15 | 29 | 55 | 0.6469 | 7 | 2 |
| Author co-occurrence | 5 | 259 | 1238 | 0.6451 | 19 | 5 |
| Author co-occurrence | 6 | 179 | 782 | 0.6359 | 18 | 8 |
| Author co-occurrence | 8 | 95 | 433 | 0.5973 | 14 | 7 |
| Keyword co-occurrence | 5 | 468 | 10800 | 0.2655 | 6 | 1 |
| Keyword co-occurrence | 6 | 356 | 8491 | 0.2401 | 6 | 1 |
| Keyword co-occurrence | 8 | 247 | 6124 | 0.2271 | 5 | 1 |

Supplementary Tables S5-S6 (provided as separate Excel files)

Table S5 (Supplementary_Table_S5_Network_Sensitivity_and_Node_Edge_Tables.xlsx): threshold-sensitivity analysis and complete node/edge tables for country, institution, author, and keyword networks.

Table S6 (Supplementary_Table_S6_Included_WOSCC_Records.xlsx): the 1,404 WOSCC records included in the bibliometric analysis.

Note: the supplied 1,677-record WOSCC export and the 1,404-record cleaned analysis set are retained by the authors. Raw WOSCC records remain subject to Clarivate licensing terms.
